# 1,3,4-Oxadiazole Derivatives: Synthesis, Characterization, Antimicrobial Potential, and Computational Studies

**DOI:** 10.1155/2014/172791

**Published:** 2014-07-24

**Authors:** Suman Bala, Sunil Kamboj, Anu Kajal, Vipin Saini, Deo Nanadan Prasad

**Affiliations:** ^1^M. M. College of Pharmacy, Maharishi Markandeshwar University, Mullana, Ambala, Haryana 133207, India; ^2^Shivalik College of Pharmacy, Nangal, Punjab 140126, India

## Abstract

We report the synthesis and biological assessment of 1,3,4-oxadiazole substituted 24 derivatives as novel, potential antibacterial agents. The structures of the newly synthesized derivatives were established by the combined practice of UV, IR, ^1^H NMR, ^13^C NMR, and mass spectrometry. Further these synthesized derivatives were subjected to antibacterial activity against all the selected microbial strains in comparison with amoxicillin and cefixime. The antibacterial activity of synthesized derivatives was correlated with their physicochemical and structural properties by QSAR analysis using computer assisted multiple regression analysis and four sound predictive models were generated with good *R* ^2^, *R*_ adj _^2^, and Fischer statistic. The derivatives with potent antibacterial activity were subjected to molecular docking studies to investigate the interactions between the active derivatives and amino acid residues existing in the active site of peptide deformylase to assess their antibacterial potential as peptide deformylase inhibitor.

## 1. Introduction

Oxadiazoles are the heterocyclic compounds containing one oxygen and two nitrogen atoms in a five membered ring [1, 2] possessing a diversity of useful biological effects [3]. Oxadiazole is considered to be resultant from furan by replacement of two methane (–CH=) groups by two pyridine type nitrogen atoms (–N=) [2]. Several methods have been reported in the literature for the synthesis of 1,3,4-oxadiazoles. The commonly used synthetic route for 1,3,4-oxadiazoles includes reactions of acid hydrazides (or hydrazine) with acid chlorides/carboxylic acids and direct cyclization of diacylhydrazines using a variety of dehydrating agents such as phosphorous oxychloride [3], thionyl chloride [4], phosphorous pentaoxide [5], triflic anhydride [6], polyphosphoric acid [7], and direct reaction of acid with (N-isocyanimino-) triphenylphosphorane [8–11].

Researchers have already reported that gram positive bacteria are much more susceptible to antimicrobial agents as compared to gram negative bacteria [12]. These differences may be attributed to the fact that the cell wall in gram positive bacteria is of single layer whereas the gram negative bacteria have multilayered cell wall. Gram negative bacteria possess an outer membrane and a unique periplasmic space which is not found in gram positive bacteria [13]. The resistance of gram negative bacteria towards antibacterial substances is due to more lipophilic nature of membrane, which acts as a barrier for various antimicrobial compounds. It was expected that hydrophilic compounds are unable to penetrate the cell membranes of these bacteria. Gram positive bacteria do not have such outer membrane and complex cell wall structure. Antibacterial substances can easily destroy the bacterial cell wall and cytoplasmic membrane of gram positive bacteria, which results in leakage of the cytoplasm [14].

Peptide deformylase (PDF) is a vital and extremely conserved enzyme, belongs to a subfamily of metalloprotease [15], and has emerged out as a target of efforts to develop novel antibacterial agents [16]. It embraces iron and is responsible for protein maturation by the exclusion of the N-formyl group from the terminal methionine residue through Fe^2+^ mediated catalysis which leads to inhibition of protein synthesis in bacteria [17].

Computational studies are the crucial steps in the drug designing. There are numerous areas of computational studies and one of them is identification of relationships between chemical structures and properties and recognized as QSAR. Quantitative structural activity relationship practices molecular parameters to enumerate a pharmacological or chemical property for a set of molecules [18].

Docking study is the computational routine to determine probable binding manners of a ligand to the dynamic site of a receptor. It makes an image of the dynamic site with interaction points known as grid. Then it fits the ligand in the binding site either by grid search or energy search [19].

In the present paper, we have explored substituted 1,3,4-oxadiazole derivatives as antibacterial agents with the support of QSAR and molecular docking studies by targeting the enzyme peptide deformylase.

## 2. Chemistry

**Figure 1:**
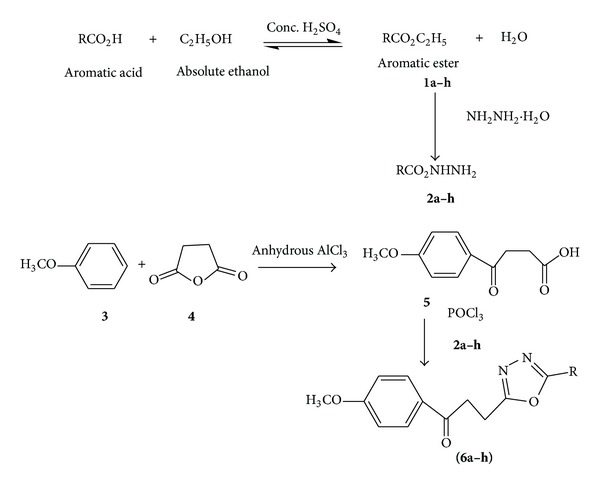
Synthetic scheme of 1-(4-methoxy-phenyl)-3-[5-(substituted phenyl)-1,3,4-oxadiazol-2-yl]propan-1-one** (6a–h)**.

**Figure 2:**
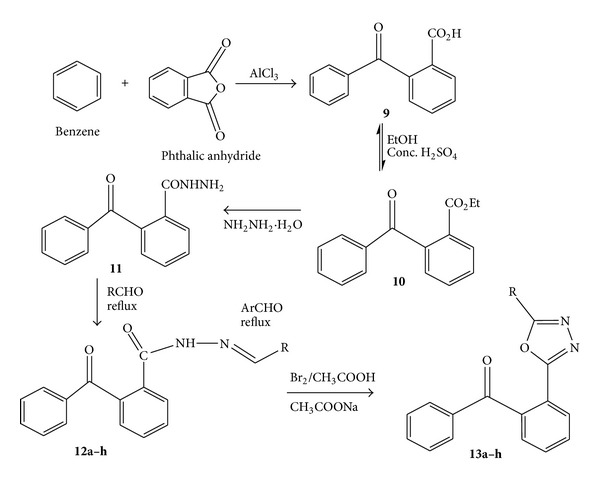
Synthetic scheme of [2-(5-substituted-phenyl-[1,3,4]oxadiazol-2-yl)-phenyl]phenyl-methanone** (13a–h)**.

The substituted aromatic acids were used as a versatile starting material for the synthesis of 1,3,4-oxadiazoles derivatives involving the formation of corresponding esters and hydrazides. Ethyl esters were synthesized from substituted aromatic acids by means of Fischer esterification which were further reacted with hydrazine hydrate in presence of ethanol to get corresponding hydrazide derivative. The hydrazide derivatives then reacted with *β*-benzoyl propionic acid in the presence of phosphorus oxychloride (cyclodehydrating agent) to get final compounds** 6a–h** (Figure 1). The chemistry of compounds** 8a–h** was already reported in our previous studies [20]. The substituted aromatic acid hydrazides from* o*-benzoyl benzoic acid reacted with aromatic aldehydes under slight acidic conditions to get the substituted hydrazone derivatives which were then cyclized in presence of bromine, acetic acid, and sodium acetate to get 1,3,4-oxadiazole derivatives** (13a–h)** (Figure 2). Reaction monitoring was done by means of thin layer chromatography (TLC). All the new synthesized compounds were characterized by melting point and spectroscopic analysis (UV-Visible, IR,^1^H NMR, ^13^C NMR, and MS).

## 3. Experimental Section

### 3.1. Material and Method

Reagent and solvents used were obtained from commercial sources. Analytical thin layer chromatography was carried out on TLC plates of 3 × 15 cm coated with silica gel G for reaction monitoring and for determination of retardation factor. Spots of TLC were located by iodine chamber. Melting points of newly synthesized derivatives were determined on digital melting point apparatus (Flora; Perfit, India) and were found uncorrected (Table 2). The *λ*_max⁡_ was calculated by using double beam UV-Visible 1800 Shimadzu spectrophotometer and the values are given in Table 2. The IR spectra were recorded on FTIR-Shimadzu spectrometer using Nujol method. ^1^H NMR and ^13^C NMR spectra were recorded on BRUKER AVANCE II 400 NMR spectrometer operating at 400 MHz and 125 MHz, respectively, using CDCl_3_, chemical shift values were expressed in *δ* ppm. For mass spectra, solutions were made in HPLC grade methanol and spectra were obtained with Vg-11-250J70S spectrophotometer at 70 eV using electron ionization (EI source). Chem 3D Ultra (version 10) was employed for structural similarity studies. QSAR studies were performed by multiple linear regression analysis using Analyze-it version 3.0 software. Molegro Virtual Docker 5.0.0. software was employed for the molecular docking studies.

### 3.2. General Procedure for the Synthesis of 1-(4-Methoxy-phenyl)-3-(5-phenyl-1,3,4-oxadiazol-2-yl)propan-1-one **(6a–h)**

Aryl hydrazide** 2a** (1 M) was dissolved in phosphorous oxychloride (5 mL) and to it compound** 5** (equimolar; 1 M) was added. The reaction mixture, after refluxing for 6-7 hours, was cooled to room temperature and poured onto crushed ice. On neutralization of the contents with sodium bicarbonate solution (20%), a solid mass separated out. This was filtered and washed with water. It was crystallized by using methanol to give** 6a**. Similarly compounds (**6b–h**) were prepared (Figure 1) [21, 22]. The corresponding R for** 6a–h** is given in Table 1.

#### 3.2.1. 1-(4-Methoxy-phenyl)-3-(5-phenyl-1,3,4-oxadiazol-2-yl)propan-1-one **(6a)**

Yield 89.90%, yellow crystals, mp (°C): 240–242; IR (Nujol, cm^−1^): 3003.17 (CH_arom_), 2818 (CH_aliph_), 1685.79 (C=O), 1595.13 (C=N), 1255.66 (C–O–C_asymm_), 1166.93 (CH bend), 1018.41 (C–O–C_symm_), 931.62, 823.60 (CH bend); ^1^H NMR (CDCl_3_, 400 MHz, ppm): 8.1 (d, 2H, H-*p-*methoxy phenyl (*J* = 7 Hz), 7.9 (d, 2H, H-2,6-phenyl (*J* = 8.1 Hz), 7.6 (d, 3H, H-3,4,5-phenyl (*J* = 7.7 Hz), 7.1 (d, 2H, H-*p-*methoxy phenyl (*J* = 7.3 Hz), 3.8 (s, 3H, OCH_3_), 3.1 (t, 2H, CH_2_ (*J* = 6.4 Hz), 2.9 (t, 2H, CH_2_ (*J* = 6.1 Hz); ^13^C NMR (CDCl_3_); 24.6 (C-6), 41.2 (C-7), 197.2 (C-8), 136.5 (C-10), 127.0 (C-11,15), 129.1 (C-12,14), 128.5 (C-13), 129.7 (C-16), 129.5 (C-17,21), 114.2 (C-18,20), 165.9 (C-19), 56.2 (C-22); MS:* m*/*z* 308.12, 309.12 (M + 1), 310.12 (M + 2).

#### 3.2.2. 1-(4-Methoxy-phenyl)-3-[5-(4-aminophenyl)-1,3,4-oxadiazol-2-yl]propan-1-one **(6b)**

Yield 83.0%, light yellow crystals, mp (°C): 242-243; IR (Nujol, cm^−1^): 3450.65 (NH), 2999.31, 2970.38 (CH), 1703.14 (C=O), 1699.29 (C=N), 1577.77 (NH), 1286.52 (C–O–C_asymm_), 1165 (CH), 1058.92 (C–O–C_symm_), 929.69, 842.69 (CH); ^1^H NMR (CDCl_3_, 400 MHz, ppm): 7.99 (d, 2H, H-*p-*methoxy phenyl (*J* = 8.4 Hz), 7.59 (d, 2H, H-*p-*amino phenyl (*J* = 8 Hz), 7.09 (d, 2H, H-*p-*methoxy phenyl (*J* = 7.8 Hz), 6.49 (d, 2H, H-*p-*amino phenyl (*J* = 7.9 Hz), 3.98 (s, 2H, NH_2_), 3.79 (s, 3H, OCH_3_), 3.10 (t, 2H, CH_2_ (*J* = 6.6 Hz), 2.89 (t, 2H, CH_2_ (*J* = 6.3 Hz); ^13^C NMR (CDCl_3_); 24.6 (C-6), 40.9 (C-7), 197.5 (C-8), 126.5 (C-10), 127.8 (C-11,15), 115.6 (C-12,14), 146.7 (C-13), 129.7 (C-16), 129.6 (C-17,21), 114.0 (C-18,20), 166.2 (C-19), 55.8 (C-23); MS:* m*/*z* 323.13, 324.12 (M + 1), 325.15 (M + 2).

#### 3.2.3. 1-(4-Methoxy-phenyl)-3-[5-(4-hydroxyphenyl)-1,3,4-oxadiazol-2-yl]propan-1-one **(6c)**

Yield 89.80%, yellow powder, mp (°C): 255.00; IR (Nujol, cm^−1^): 3653.18 (OH), 3045.60 (CH_arom_), 2767.85 (CH_aliph_), 1670.35 (C=O), 1602.85 (C=N), 1543.06 (C=C), 1365.60 (OH), 1282.66 (C–O–C_asymm_), 1031.92 (C–O–C_symm_), 767.67 (CH); ^1^H NMR (CDCl_3_, 400 MHz, ppm): 7.9 (d, 2H, H-*p-*methoxy phenyl (*J* = 7.8 Hz), 7.7 (d, 2H,H-*p-*hydroxy phenyl (*J* = 7.7 Hz), 7.0 (d, 2H, H-*p-*methoxy phenyl (*J* = 8.0 Hz), 6.8 (d, 2, H-*p-*hydroxy phenyl (*J* = 8.0 Hz), 4.9 (s, 1H,OH), 3.7 (s, 3H,OCH_3_), 3.0 (t, 2H,CH_2_ (*J* = 6.1 Hz), 2.8 (t, 2H,CH_2_ (*J* = 6.0 Hz); ^13^C NMR (CDCl_3_); 24.7 (C-6), 40.6 (C-7), 197.6 (C-8), 129.1 (C-10), 128.4 (C-11,15), 116.2 (C-12,14), 157.3 (C-13), 129.7 (C-16), 129.6 (C-17,21), 114.0 (C-18,20), 166.5 (C-19), 56.0 (C-23); MS:* m*/*z* 324.11, 325.13 (M + 1), 326.12 (M + 2).

#### 3.2.4. 1-(4-Methoxy-phenyl)-3-[5-(2-hydroxyphenyl)-1,3,4-oxadiazol-2-yl]propan-1-one **(6d)**

Yield 80.80%, yellow crystals, mp (°C): 253.00; IR (Nujol, cm^−1^): 3626.33 (OH), 3025.48, 2925.17 (CH), 1698.40 (C=O), 1605.81 (C=N), 1522.87 (C=C), 1363.73 (OH), 1259.57 (C–O–C_asymm_), 1148.66 (CH), 1013.64 (C–O–C_symm_), 895.99, 817.85, 738.77 (CH); ^1^H NMR (CDCl_3_, 400 MHz, ppm): 7.9 (d, 2H, H-*p-*methoxy phenyl (*J* = 8.4 Hz), 7.6 (m, 1H, H-*p-*hydroxy phenyl (*J* = 7.6 Hz), 7.3 (m, 1H, H-*o-*hydroxy phenyl (*J* = 7.7 Hz), 7.1 (m, 3H, 2H-*p-*methoxy phenyl and 1H-*o-*hydroxy phenyl), 6.9 (m, 1H, H-*o-*hydroxy phenyl (*J* = 8.1 Hz), 4.4 (s, 1H, OH), 3.8 (s, 3H, OCH_3_), 3.1 (t, 2H, CH_2_ (*J* = 6.1 Hz), 2.9 (t, 2H, CH_2_ (*J* = 6.0 Hz) ^13^C NMR (CDCl_3_); 24.4 (C-6), 40.9 (C-7), 197.4 (C-8), 123.7 (C-10), 155.8 (C-11), 116.2 (C-12), 129.9 (C-13), 121.6 (C-14), 128.4 (C-15), 129.6 (C-16), 129.4 (C-17,21), 114.1 (C-18,20), 166.4 (C-19), 56.2 (C-23); MS:* m*/*z* 324.11, 325.13 (M + 1), 326.12 (M + 2).

#### 3.2.5. 1-(4-Methoxy-phenyl)-3-[5-(4-chlorophenyl)-1,3,4-oxadiazol-2-yl]propan-1-one **(6e)**

Yield 78.80%, yellow powder, mp (°C): 270–272; IR (Nujol, cm^−1^): 3064.89 (CH_arom_), 2742.76 (CH_aliph_), 1614.42 (C=O), 1539.29 (C=N), 1292.24 (C–O–C_asymm_), 1018.41 (C–O–C_symm_), 796.53 (CH), 567.07 (C–Cl); ^1^H NMR (CDCl_3_, 400 MHz, ppm): 7.9 (d, 2H,* p-*methoxy phenyl (*J* = 7.68 Hz), 7.7 (d, 2H,* p-*chloro phenyl (*J* = 7.7 Hz), 7.5 (d, 2H,* p-*chloro phenyl (*J* = 8.0 Hz), 7.1 (d, 2H,* p-*methoxy phenyl (*J* = 8.1 Hz), 3.8 (s, 3H, OCH_3_), 3.2 (t, 2H, CH_2_ (*J* = 6.1 Hz), 3.0 (t, 2H, CH_2_ (*J* = 6.0 Hz); ^13^C NMR (CDCl_3_); 24.4 (C-6), 40.8 (C-7), 198.0 (C-8), 134.6 (C-10), 128.4 (C-11,15), 129.4 (C-12,14), 133.8 (C-13), 129.7 (C-16), 129.7 (C-17,21), 114.0 (C-18,20), 166.8 (C-19), 56.1 (C-23); MS:* m*/*z* 342.08, 344.07 (M + 1), 343.08 (M + 2).

#### 3.2.6. 1-(4-Methoxy-phenyl)-3-[5-(2-chlorophenyl)-1,3,4-oxadiazol-2-yl]propan-1-one **(6f)**

Yield 78.00%, Yellow powder, mp (°C): 273.0; IR (Nujol, cm^−1^): 3076.46 (CH_arom_), 2744.71 (CH_aliph_), 1618.28 (C=O), 1556.55 (C=N), 1051.20 (C–O–C), 821.68 (CH), 590.22 (C–Cl); ^1^H NMR (CDCl_3_, 400 MHz, ppm): 8.0 (d, 2H,H-*p-*methoxy phenyl (*J* = 8.9 Hz), 7.6 (m, 2H,H-*o-*chloro phenyl (*J* = 8.1 Hz), 7.4 (m, 1H, H-*o-*chloro phenyl), 7.2 (m, 1H, H-*o-*chloro phenyl), 7.1 (d, 2H, H-*p-*methoxy phenyl (*J* = 8.2 Hz), 3.7 (s, H, OCH_3_), 3.2 (t, H, CH_2_ (*J* = 6.3 Hz), 3.0 (t, H, CH_2_ (*J* = 6.3 Hz); ^13^C NMR (CDCl_3_); 24.2 (C-6), 40.4 (C-7), 196.9 (C-8), 137.9 (C-10), 127.4 (C-11), 134.3 (C-12), 128.9 (C-13), 130.4 (C-14), 125.1 (C-15), 128.9 (C-16), 129.5 (C-17,21), 114.0 (C-18,20), 166.9 (C-19), 56.4 (C-23); MS:* m*/*z* 342.08, 344.07 (M + 1), 343.08 (M + 2).

#### 3.2.7. 1-(4-Methoxy-phenyl)-3-[5-(4-methylphenyl)-1,3,4-oxadiazol-2-yl]propan-1-one **(6g)**

Yield 79.20%, Brown crystals, mp (°C): 250–253; IR (Nujol, cm^−1^): 3028.24 (CH_arom_), 2779.42 (CH_aliph_), 1699.29 (C=O), 1514.12 (C=N), 1301.95 (C–O–C_asymm_), 1190.08 (C–O–C_symm_), 908.47, 819.75, 773.46 (CH); ^1^H NMR (CDCl_3_, 400 MHz, ppm): 7.8 (d, 2H, H-*p-*methoxy phenyl (*J* = 8.4 Hz), 7.5 (d, 2H, H-*p-*methyl phenyl (*J* = 8.4 Hz), 7.2 (d, 2H, H-*p-*methyl phenyl (*J* = 8.3 Hz), 7.0 (d, 2H, H-*p-*methoxy phenyl (*J* = 8.4 Hz), 3.7 (s, 3H, OCH_3_), 3.1 (t, 2H, CH_2_), 2.9 (t, 2H, CH_2_ (*J* = 6.2 Hz), 2.3 (s, 3H, CH_3_ (*J* = 6.0 Hz); ^13^C NMR (CDCl_3_); 24.9 (C-6), 41.2 (C-7), 197.4 (C-8), 133.5 (C-10), 126.6 (C-11,15), 129.3 (C-12,14), 137.7 (C-13), 129.2 (C-16), 129.4 (C-17,21), 20.9 (C-22), 114.2 (C-18,20), 166.5 (C-19), 56.0 (C-23); MS:* m*/*z* 322.13, 323.14 (M + 1), 324.11 (M + 2).

#### 3.2.8. 1-(4-Methoxy-phenyl)-3-[5-(4-nitrophenyl)-1,3,4-oxadiazol-2-yl]propan-1-one **(6h)**

Yield 90.0%, yellow crystals, mp (°C): 250.00; IR (Nujol, cm^−1^): 3024.38, 2906 (CH), 1662.64 (C=O), 1631.78 (C=N), 1587.42 (C=C), 1517.98 (N=O_asymm_), 1317.38 (N=O_symm_), 1064.71 (C–O–C), 939.33, 742 (CH); ^1^H NMR (CDCl_3_, 400 MHz, ppm): 8.4 (d, 2H, H-*p-*nitro phenyl (*J* = 8.0 Hz), 8.2 (d, 2H, H-*p-*methoxy phenyl (*J* = 7.7 Hz), 7.8 (d, 2H, H-*p-*nitro phenyl (*J* = 7.8 Hz), 7.0 (d, 2H, H-*p-*methoxy phenyl (*J* = 7.6 Hz), 3.7 (s, 3H, OCH_3_), 3.1 (t, 2H, CH_2_ (*J* = 6.3 Hz), 2.9 (t, 2H, CH_2_ (*J* = 6.3 Hz); ^13^C NMR (CDCl_3_); 24.4 (C-6), 40.9 (C-7), 197.6 (C-8), 142.6 (C-10), 127.9 (C-11,15), 124.1 (C-12,14), 148.4 (C-13), 129.7 (C-16), 129.6 (C-17,21), 114.0 (C-18,20), 166.4 (C-19), 56.0 (C-23); MS:* m*/*z* 353.10, 354.11 (M + 1), 355.11 (M + 2).

### 3.3. General Procedures for the Synthesis of [2-(5-Substituted-phenyl-[1,3,4]oxadiazol-2-yl)-phenyl]-phenyl-methanone **(13a–h)**

A mixture of** 12a** (0.01 M), anhydrous sodium acetate (0.02 M), and glacial acetic acid was placed in round bottomed flask equipped with separating funnel for the addition of bromine. Bromine (0.8 mL in 5 mL of glacial acetic acid) was added slowly to it while stirring magnetically. After 2 hours of stirring the solution was poured on crushed ice. The resulting solid was separated, dried, and recrystallized from ethyl alcohol. Similarly compounds (**13b–h**) were prepared (Figure 2) [23]. The corresponding R for** 13a–h** is given in Table 1.

#### 3.3.1. {2-[5-(4-Phenyl)-[1,3,4]oxadiazol-2-yl]-phenyl}-phenyl-methanone **(13a)**

Yield 72.45%, white crystals, mp (°C): 291.00; IR (Nujol, cm^−1^): 3030.17 (CH), 1627.92 (C=O), 1600.92 (C=N), 1519.91 (C=C), 1219.09 (C–O–C_asymm_), 1043.49 (C–O–C_symm_), 920.05, 779.24, 738.74 (CH); ^1^H NMR (CDCl_3_, 400 MHz, ppm): 8.3 (d, 1H, H-diphenyl methanone (*J* = 8.4 Hz), 7.9 (d, 2H, H-phenyl (*J* = 7.7 Hz), 7.7 (m, 3H, H-diphenyl methanone (*J* = 7.7 Hz), 7.6 (m, 1H, H-diphenyl methanone), 7.5 (m, 5H, 2H-diphenyl methanone and 3H-phenyl), 7.3 (m, 2H, diphenyl methanone); ^13^C NMR (CDCl_3_): 136.2 (C-6), 128.6 (C-7), 138.3 (C8), 130.1 (C-9), 128.7 (C-10), 130.7 (C-11), 136.5 (C-12), 127.0 (C-13,17), 129.0 (C-14,16), 128.5 (C-15), 187.0 (C-18), 137.8 (C-20), 130.1 (C-21,25), 128.2 (C-22,24), 132.2 (C-23); MS:* m*/*z* 326.14, 326.11 (M + 1), 327.11 (M + 2).

#### 3.3.2. {2-[5-(4-Chlorophenyl)-[1,3,4]oxadiazol-2-yl]-phenyl}-phenyl-methanone **(13b)**

Yield 76.60%, yellow powder, mp (°C): 283–285; IR (Nujol, cm^−1^): 3034.03, 2918.30 (CH), 1683.86 (C=O), 1583.56 (C=N), 1521.84 (C=C), 1257.59 (C–O–C_asymm_), 1176.58 (CH), 1058.92 (C–O–C_symm_), 925.83, 811.78, 756.10 (CH), 545.85 (C–Cl); ^1^H NMR (CDCl_3_, 400 MHz, ppm): 8.2 (d, 1H, H-diphenyl methanone) (*J* = 7.8 Hz), 7.8 (m, 3H,H-diphenyl methanone and 2H-*p-*chlorophenyl), 7.6 (m, 3H,H-diphenyl methanone), 7.4 (m, 2H, diphenyl methanone (*J* = 7.8 Hz), 7.2 (d, 4H, 2H-diphenyl methanone and 2H-*p-*chlorophenyl); ^13^C NMR (CDCl_3_): 136.4 (C-6), 128.5 (C-7), 138.7 (C-8), 130.5 (C-9), 128.2 (C-10), 130.9 (C-11), 134.6 (C-12), 128.4 (C-13,17), 129.4 (C-14,16), 133.8 (C-15), 187.2 (C-18), 137.4 (C-21), 130.6 (C-22,26), 128.8 (C-23,25), 131.9 (C-24); MS:* m*/*z* 360.07, 362.06 (M + 1), 363.10 (M + 2).

#### 3.3.3. {2-[5-(4-Nitrophenyl)-[1,3,4]oxadiazol-2-yl]-phenyl}-phenyl-methanone **(13c)**

Yield 75.50%, brown crystals, mp (°C): 261–263; IR (Nujol, cm^−1^): 3115.04, 2920.23 (CH), 1651.07 (C=O), 1620.21 (C=N), 1571.99 (C=C), 1496.76 (N=O_asymm_), 1365.60 (N=O_symm_), 1209.37 (C–O–C_asymm_), 1120.64 (CH), 1047.35 (N=O_symm_), 798.53 (CH); ^1^H NMR (CDCl_3_, 400 MHz, ppm): 8.6 (d, 2H, H-*p-*nitrophenyl (*J* = 7.7 Hz), 8.3 (m, 3H, 1H-diphenyl methanone and 2H-*p-*nitrophenyl), 7.9 (m, 3H, 1H-diphenyl methanone (*J* = 8.0 Hz), 7.7 (m, 3H, 1H-diphenyl methanone (*J* = 7.7 Hz), 7.4 (m, 2H, 1H-diphenyl methanone (*J* = 7.9 Hz); ^13^C NMR (CDCl_3_): 136.0 (C-6), 128.2 (C-7), 138.3 (C-8), 130.9 (C-9), 128.0 (C-10), 130.4 (C-11), 142.6 (C-12), 127.9 (C-13,17), 124.1 (C-14,16), 148.4 (C-15), 186.9 (C-18), 137.9 (C-21), 128.2 (C-22,26), 127.2 (C-23,25), 129.9 (C-24); MS:* m*/*z* 371.09, 372.06 (M + 1), 373.10 (M + 2).

#### 3.3.4. {2-[5-(4-Methylphenyl)-[1,3,4]oxadiazol-2-yl]-phenyl}-phenyl-methanone **(13d)**

Yield 75.50%, yellow powder, mp (°C): 257–259; IR (Nujol, cm^−1^): 3000 (CH), 1655.96 (C=O), 1563.337 (C=N), 1521.90 (C=C), 1236.42 (C–O–C_asymm_), 1111.05 (CH), 1041.61 (C–O–C_symm_), 858.36, 820.75 (CH); ^1^H NMR (CDCl_3_, 400 MHz, ppm): 8.5 (d, 1H, 1H-diphenyl methanone (*J* = 7.8 Hz), 8.0 (d, 2H,* p-*methyl phenyl (*J* = 8.0 Hz), 7.8 (m, 3H, diphenyl methanone (*J* = 8.0 Hz), 7.6 (m, 3H, diphenyl methanone (*J* = 7.9 Hz), 7.5 (m, 2H, diphenyl methanone (*J* = 7.6 Hz), 7.1 (d, 2H,* p-*methyl phenyl (*J* = 7.7 Hz), 2.4 (s, 3H, CH_3_); ^13^C NMR (CDCl_3_): 136.9 (C-6), 128.2 (C-7), 138.7 (C-8), 130.5 (C-9), 128.2 (C-10), 130.9 (C-11), 133.5 (C-12), 126.9 (C-13,17), 129.7 (C-14,16), 137.7 (C-15), 187.2 (C-18), 20.9 (C-20), 137.4 (C-21), 130.6 (C-22,26), 128.8 (C-23,25), 131.9 (C-24); MS:* m*/*z* 340.10, 341.12 (M + 1), 342.11 (M + 2).

#### 3.3.5. {2-[5-(3-Methoxy-4-hydroxyphenyl)-[1,3,4]oxadiazol-2-yl]-phenyl}-phenyl-methanone **(13e)**

Yield 78.80%, yellow powder, mp (°C): 268–271; IR (Nujol, cm^−1^): 3605.11 (OH), 3073.70, 3032.23 (CH), 1662.71 (C=O), 1558.55 (C=N), 1510.33 (C=C), 1310.69 (OH), 1254.75 (C–O–C), 1117.80 (CH), 983.74, 835.21 (CH); ^1^H NMR (CDCl_3_, 400 MHz, ppm): 8.4 (d, 1H, H-diphenyl methanone (*J* = 7.9 Hz), 7.9 (m, 3H, H-diphenyl methanone (*J* = 7.6 Hz), 7.75 (m, 3H, H-diphenyl methanone (*J* = 7.7 Hz), 7.4 (m, 3H, 1H-*p-*hydroxy-3-methoxyphenyl and 2H-diphenyl methanone), 7.2 (s, 1H, 1H-*p-*hydroxy-3-methoxyphenyl), 6.9 (d, 1H, 1H-*p-*hydroxy-3-methoxypheny l (*J* = 7.5 Hz), 5.5 (s, 1H,OH), 3.7 (s, 3H,OCH_3_); ^13^C NMR (CDCl_3_): 130.1 (C-6), 114.0 (C-7), 149.7 (C-8), 142.9 (C-9), 117.2 (C-10), 120.7 (C-11), 136.8 (C-12), 127.9 (C-13), 138.0 (C-14), 129.4 (C-15), 128.4 (C-16), 130.1 (C-17), 56.3 (C-18), 188.1 (C-20), 137.8 (C-21), 129.9 (C-23,27), 128.5 (C-24,26), 131.9 (C-25); MS:* m*/*z* 372.11, 373.13 (M + 1), 374.12 (M + 2).

#### 3.3.6. {2-[5-(4-Hydroxyphenyl)-[1,3,4]oxadiazol-2-yl]-phenyl}-phenyl-methanone **(13f)**

Yield 80.0%, light yellow powder, mp (°C): 294-295; IR (Nujol, cm^−1^): 3624.25 (OH), 3024.38 (CH), 1667.14 (C=O), 1589.34 (C=N), 1554.63 (C=C), 1402.25 (OH), 1247.94 (C–O–C), 1126.43 (CH), 933.70, 862.18, 769.60 (CH); ^1^H NMR (CDCl_3_, 400 MHz, ppm): 8.3 (d, 1H, H-diphenyl methanone (*J* = 8.1 Hz), 7.9 (m, 5H, 2H-4-hydroxy phenyl, 3H-diphenyl methanone), 7.6 (m, 3H, H-diphenyl methanone (*J* = 7.8 Hz), 7.39 (m, 2H, H-diphenyl methanone (*J* = 7.8 Hz), 7.3 (m, 2H, H-diphenyl methanone (*J* = 8.1 Hz), 6.9 (d, 2H,H-*p-*hydroxy phenyl (*J* = 7.8 Hz), 5.09 (s, 1H,OH); ^13^C NMR (CDCl_3_): 136.9 (C-6), 128.2 (C-7), 138.7 (C-8), 130.5 (C-9), 128.2 (C-10), 130.9 (C-11), 129.1 (C-12), 128.4 (C-13,17), 116.2 (C-14,16), 157.3 (C-15), 187.2 (C-18), 20.9 (C-20) 137.4 (C-21), 130.6 (C-22,26), 128.8 (C-23,25), 131.9 (C-24); MS:* m*/*z* 342.10, 343.18 (M + 1), 344.11 (M + 2).

#### 3.3.7. {2-[5-(2-Nitrophenyl)-[1,3,4]oxadiazol-2-yl]-phenyl}-phenyl-methanone **(13g)**

Yield 75.85%, yellow crystals, mp (°C): 237–239; IR (Nujol, cm^−1^): 2920.35 (CH), 1668.50 (C=O), 1522.87 (C=N), 1518.04 (C=C), 1511.29 (N=O_asymm_), 1371.45 (N=O_symm_), 1265.36 (C–O–C), 1160.23, 1108.15, 1098.51 (CH), 758.06 (CH); ^1^H NMR (CDCl_3_, 400 MHz, ppm): 8.3 (d, 1H, H-diphenyl methanone (*J* = 8.2 Hz), 8.0 (m, 2H,* o-*nitrophenyl (*J* = 7.7 Hz), 7.7 (m, 4H, 1H-*o-*nitrophenyl and 3H-diphenyl methanone), 7.5 (m, 2H, 1H-o-nitrophenyl and 1H-diphenyl methanone), 7.4 (m, 4H, H-diphenyl methanone (*J* = 7.8 Hz); ^13^C NMR (CDCl_3_): 131.6 (C-6), 146.9 (C-7), 124.1 (C-8), 129.4 (C-9), 135.1 (C-10), 127.9 (C-11), 135.9 (C-12), 127.9 (C-13), 138.0 (C-14), 129.4 (C-15), 128.4 (C-16), 130.1 (C-17), 187.0 (C-19), 137.4 (C-21), 130.6 (C-22,26), 128.8 (C-23,25), 131.9 (C-24); MS:* m*/*z* 371.10, 372.09 (M + 1), 373.10 (M + 2).

#### 3.3.8. {2-[5-(2-Hydroxyphenyl)-[1,3,4]oxadiazol-2-yl]-phenyl}-phenyl-methanone **(13h)**

Yield 78.0%, yellow crystals, mp (°C): 254-255; IR (Nujol, cm^−1^): 3381.21 (OH), 2987.74 (CH), 1602.85 (C=O), 1591.27 (C=N), 1352.10 (OH), 1070.49 (C–O–C), 742.59 (CH); ^1^H NMR (CDCl_3_, 400 MHz, ppm): 8.5 (s, 1H, OH), 8.39 (d, 1H, H-diphenyl methanone (*J* = 8.5 Hz), 7.81 (m, 3H, H-diphenyl methanone (*J* = 7.5 Hz), 7.61 (m, 5H, H-diphenyl methanone and* o-*hydroxy phenyl), 7.3 (m, 1H, H-diphenyl methanone (*J* = 8.2 Hz), 6.8 (m, 1H,* o-*hydroxy phenyl (*J* = 7.7 Hz), 6.7 (m, 1H,* o-*hydroxy phenyl (*J* = 7.6 Hz), 6.5 (m, 1H,* o-*hydroxy phenyl (*J* = 7.7 Hz); ^13^C NMR (CDCl_3_): 123.7 (C-6), 155.8 (C-7), 116.2 (C-8), 129.9 (C-9), 121.6 (C-10), 128.4 (C-11), 135.9 (C-12), 127.9 (C-13), 138.0 (C-14), 129.4 (C-15), 128.4 (C-16), 130.1 (C-17), 187.0 (C-19), 137.4 (C-21), 130.6 (C-22,26), 128.8 (C-23,25), 131.9 (C-24); MS:* m*/*z* 342.10, 343.12 (M + 1), 344.11 (M + 2).

## 4. Antibacterial Evaluation

### 4.1. Antibacterial Activity

The antibacterial evaluation was carried out by using agar cup-plate method. The microorganisms* Escherichia coli*,* Pseudomonas aeruginosa*,* Staphylococcus epidermidis*, and* Staphylococcus aureus* were used for the antibacterial evaluation. Nutrient agar media were prepared and then inoculated with fresh prepared culture media. The inoculated media were poured into Petri dish and allowed to set. Cups were made by punching the agar surface with a sterile cork bore (8 mm). Solutions containing 10, 12.5, 25, 50, 100, 200, 400, 800, and 1600 *μ*g/mL of the test derivatives in dimethyl formamide (DMF) were added to each cup. Amoxicillin and cefixime were taken as positive control and DMF was taken as blank. The plates were incubated at 37°C for 24 hours and the results were recorded. The zones of inhibition of the microbial growth produced by different concentration of test compounds were measured in millimeters [24, 25]. The zone of inhibition data for antibacterial compounds is summarized in Table 3.

### 4.2. Minimum Inhibitory Concentration (MIC)

**Figure 3:**
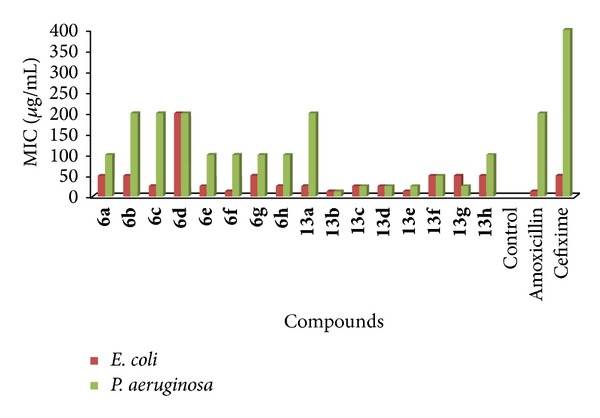
Minimum inhibitory concentrations (MIC) of synthesized 1,3,4-oxadiazole derivatives and standard drugs against gram negative bacterial strains.

**Figure 4:**
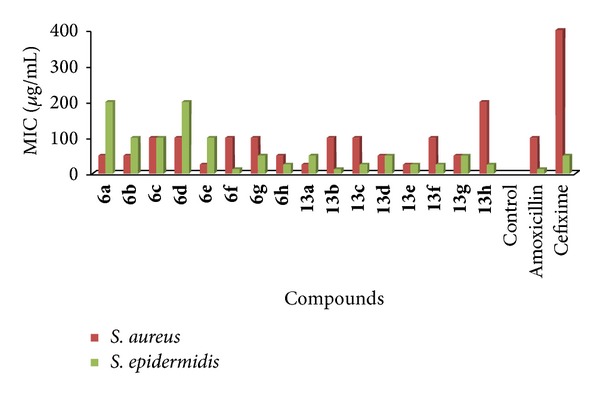
Minimum inhibitory concentrations (MIC) of synthesized 1,3,4-oxadiazole derivatives and standard drugs against gram positive bacterial strains.

Nutrient agar was prepared, sterilized, and cooled to 45°C with gentle shaking to bring about uniform cooling. It was inoculated with 0.5-0.6 mL of culture and mixed well by gentle shaking before pouring into the sterilized petri dishes. The poured materials were allowed to set and thereafter the cups were made by punching into the agar surface with sterile cork borer and scooping out the punched part of the agar. 0.1 mL of each test compounds was added into the cups with the help of sterile syringe. Twofold diluted solutions of the compounds and reference drugs were used (6.5, 12.5, 25, 50, 100, 200, 400, 800, and 1600 *μ*g/mL). The drug solutions were allowed to diffuse for some time into the medium. The plates were incubated at 30–35°C for 24–48 hours. The incubation chamber was kept sufficiently humid. MIC values were determined at the end of the incubation period. The MIC values for synthesized compounds are summarized in Table 4 and graphical representation of MIC of synthesized 1,3,4-oxadiazole derivatives and standard drugs against gram negative and gram positive bacterial strains are given in Figures 3 and 4 (Table 4), respectively [24, 25].

### 4.3. QSAR Analysis

The compounds were analyzed by multiple regression analysis, a QSAR approach using different physicochemical parameters as independent and biological activity as dependent variables [26]. Multiple linear regression efforts to maximize the fit of the data to a QSAR model for the biological activity by correcting each of the existing parameters. Successive regression models will be generated in which parameters will be either added or removed until the maximum *r* ^2^ and minimum S are obtained. The extent of coefficients obtained in this routine specifies the virtual contribution of the associated parameters to biological activity.

Biological activity data was converted to the logarithmic value. The biological activities used in the present studies were expressed as pMIC_50_; logarithm of a reciprocal concentration for 50% inhibition where MIC_50_ is the minimum inhibitory concentration of the compounds producing 50% reduction in the effect caused by bacteria (*Escherichia coli, Pseudomonas aeruginosa, Staphylococcus epidermidis*, and* Staphylococcus aureus*) is stated as a mean of at least three experiments for 24 compounds [26, 27].

The physicochemical parameters were computed (Table 5) using Chem 3D Ultra 10 after energy minimization to minimum root mean square (RMS) gradient of 0.100 kcal/mole Å by MOPAC software package. The parameters Log *P*, SAS, MR, ovality, MSA, and MW were selected in QSAR studies which are having significant effect on biological activity.

### 4.4. Molecular Docking Studies

Docking studies reported here IN were performed using software Molegro Virtual Docker 5.0.0.

The newly synthesized 1,3,4-oxadiazoles derivatives were computationally designed and optimized to investigate the interactions between the target compounds and the amino acid residues of the* E. coli* PDF.Ni enzyme by molecular docking [15]. Target compounds were docked into active site of PDF (PDB code 1G2A) using MVD (version 5.0.0.) software to observe the affinity for the enzyme and also compared their binding energies with standard drugs such as amoxicillin and cefixime.

## 5. Results and Discussion

### 5.1. Chemistry

The structures of all the newly synthesized derivatives were confirmed by chromatographic and spectroscopic (IR, ^1^H-NMR, ^13^C NMR, and mass) methods. Both analytical and spectral data of all the synthesized derivatives were in full agreement with the proposed structures. The characteristic C=N band (1680–1520 cm^−1^) of medium intensity and a medium-strong band at 1300–1050 cm^−1^ were identified in each IR spectra; the latter could be attributed to the C–O–C vibration or heteroatom ring deformation of the oxadiazole ring. ^1^H NMR showed characteristic doublets at (7.8–8.2) and 7.0-7.1) due to aromatic protons of* p-*methoxy phenyl for derivatives (**6a–h)**. The presence of two methylene groups was confirmed by presence of doublets at (3.0–3.2) and (2.8–3.0). All the other aliphatic and aromatic protons were observed within the expected regions. ^13^C data also supported the structures of the synthesized derivatives. In the mass spectra, molecular ions of medium intensity and the base peak usually belonged to the corresponding acylium ions and nitrile radical ions, which are formed by the cleavage of heteroatom ring. Both of these have been reported as a characteristic for this family. This part concluded the synthesis of 1,3,4-oxadiazole derivatives.

### 5.2. Antibacterial Activity

The antibacterial activity of the synthesized derivatives was carried out by agar cup plate method and the average radius of zone of inhibition (mm) and MIC (*μ*g/mL) were recorded in comparison with amoxicillin and cefixime. The zone of inhibition of** 6e** (27.82 ± 0.16, 20.63 ± 0.16, 24.6 ± 0.11, and 25.86 ± 0.17 at 400 *μ*g/mL),** 8h (**28.97 ± 0.16, 23.9 ± 0.1, 26.04 ± 0.14, and 26.91 ± 0.19 at 1600 *μ*g/mL),** 13b** (28.95 ± 0.18, 18.11 ± 0.11, 26.16 ± 0.18, and 26.9 ± 0.14 at 200 *μ*g/mL),** 13c** (28.15 ± 0.18, 22.13 ± 0.15, 27.08 ± 0.24, and 28 ± 0.08 at 200 *μ*g/mL), and** 13e** (28.01 ± 0.18, 22.17 ± 0.2, 28.34 ± 0.24, and 28.08 ± 0.07 at 200 *μ*g/mL) was found to be good which is comparable with zone of inhibition of amoxicillin (30 ± 0.1, 13.99 ± 0.23, 18.12 ± 0.12, and 32.06 ± 0.22 at 800 *μ*g/mL) and cefixime (22.18 ± 0.22, 12 ± 0.38, 12.03 ± 0.19, and 20.03 ± 0.15 at 800 *μ*g/mL) indicated that these derivatives have potent antibacterial activity. No inhibitory effect was observed for DMF. All the derivatives were found to be active against* Pseudomonas aeruginosa* and* Staphylococcus aureus.* The zone of inhibition and MIC and zone of inhibition of all the synthesized derivatives is summarized in Table 3 (Figure 3) and Table 4 (Figure 4), respectively.

An introspection of the active compounds revealed that different structural features of the compounds have more influence on biological activity. Three starting materials which have been selected for design and development of novel substituted 1,3,4-oxadiazole derivatives include methoxy benzene (anisole), N-phenyl anthranilic acid, and* o-*benzoyl benzoic acid.

- All these have a significant influence on increase of lipophilicity, which is responsible for the better activity of the tested compounds, may be due to easy penetration of compounds in the microbial cell membrane. The inclusion of an oxadiazole moiety in the synthesized compounds also showed high lipophilicity, hypothesizing that this lipophilicity could facilitate passage of these compounds through the bacterial membrane. All synthesized compounds have significant logP (octanol-water partition coefficient) which affect drug absorption, distribution, bioavailability, drug-receptor interactions, metabolism, and toxicity profile.
- At one side of 1,3,4-oxadiazole nucleus substitution with phenyl ring or bulkier aromatic groups may also offer significant increase in the biological activity.
- The antimicrobial activity results indicated that the presence of electron-withdrawing halogen groups and nitro group at para and ortho position of the phenyl ring improved their biological activity. On the basis of biological data, improvement in biological activity was observed with an increase in electronegativity of the molecule due to the presence of halogen group** (6e, 6f, 8e, 8f,** and** 13b)** and nitro group** (6h, 8h, 13c,** and** 13g)**.

### 5.3. QSAR Studies

Multilinear regression is one of the vital method of quantitative structure activity relationship which is used for displaying linear correlation among a dependent variable *Y* (biological activity; MIC_50_) and independent variable *X* (physicochemical parameters).

#### 5.3.1. QSAR Model for Antibacterial Activity against* Escherichia coli*

Consider the following:

(1)pMIC50=− 0.03664(Log P)+0.06895(SAS) +0.09532(MR)−44.89(Ovality) −0.06354(MSA)−0.0167(MW)+48.31,(2)N=16,  R2=0.831,Radj2=0.718,  Press=0.244,Q2=0.8304,  F=7.36,  S=0.164.

Here and hereafter, *R* ^2^: coefficient of correlation, *R*_adj_^2^: coefficient of determination, *F*: Fischer statistics, *N*: numbers of synthesized derivatives, *S*: standard error of estimate, Press: predictive error sum of squares, *Q* ^2^: cross validated *R* ^2^, and BA: biological activity.

**Figure 5:**
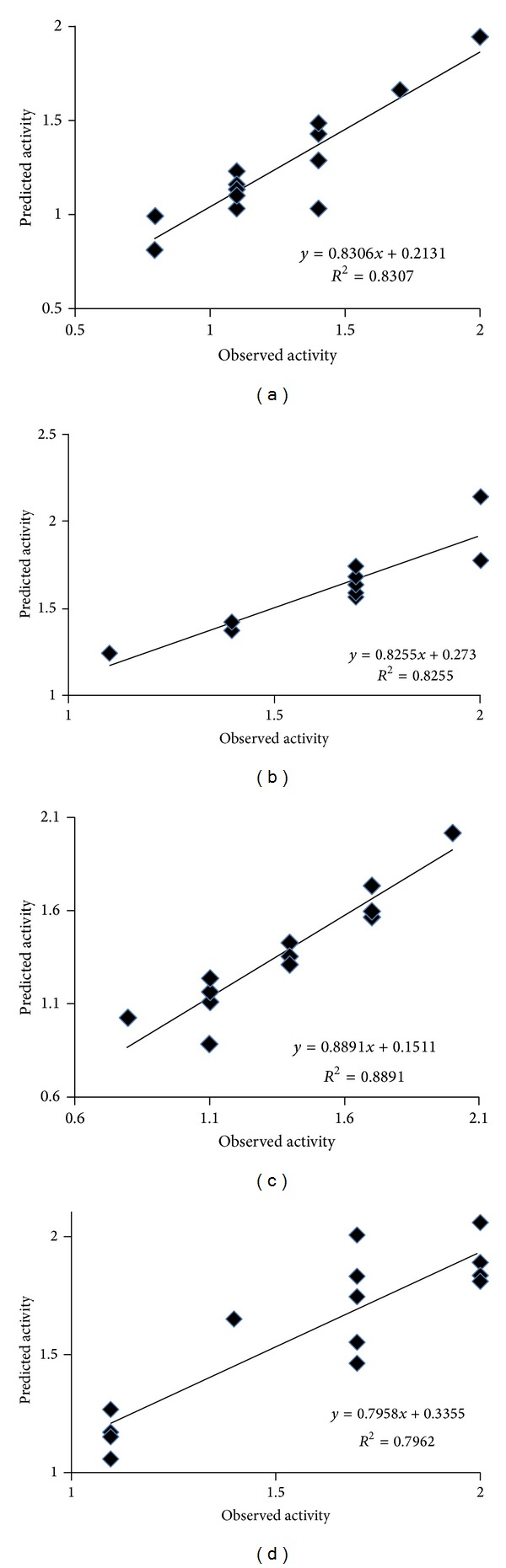
Plot of calculated pMIC_50_ values against observed pMIC_50_ for QSAR model for (a)* Escherichia coli* (b),* Pseudomonas aeruginosa* (c),* Staphylococcus aureus,* and (d)* Staphylococcus epidermidis.*

The observed and predicted antibacterial activity of synthesized 1,3,4-oxadiazole derivatives against* Escherichia coli* are summarized in Table 6 and plot of observed and predicted antibacterial activity is given in Figure 5.

#### 5.3.2. QSAR Model for Antibacterial Activity against* Pseudomonas aeruginosa*

Consider the following:

(3)pMIC50=−0.4431(Log P)−0.05285(SAS)−0.01438(MR)−3.004(Ovality)+0.1083(MSA)+0.01097(MW)+2.893,(4)N=12,  R2=0.825,Radj2=0.616,  Press=0.131,Q2=0.823,  F=3.94,  S=0.161.

The observed and predicted antibacterial activities of synthesized 1,3,4-oxadiazole derivatives against* Pseudomonas aeruginosa* are summarized in Table 6 and plot of observed and predicted antibacterial activity is given in Figure 5.

#### 5.3.3. QSAR Model for Antibacterial Activity against* Staphylococcus aureus*

Consider the following:

(5)pMIC50=0.007502(Log P)−0.01653(SAS) −0.0813(MR)−8.436(Ovality) +0.07769(MSA)−0.01452(MW)+13.04,(6)N=16,  R2=0.889,Radj2=0.815,  Press=0.170,Q2=0.8887,  F=12.02,  S=0.137.

The observed and predicted antibacterial activities of synthesized 1,3,4-oxadiazole derivatives against* Staphylococcus aureus* are summarized in Table 7 and plot of observed and predicted antibacterial activities is given in Figure 5.

#### 5.3.4. QSAR Model for Antibacterial Activity against* Staphylococcus epidermidis*

Consider the following:

(7)pMIC50=− 0.2393(Log P)+0.03799(SAS) +0.004048(MR)−6.152(Ovality) −0.07843(MSA)+0.02144(MW)+6.999,(8)N=16,  R2=0.796,Radj2=0.654,  Press=0.394,Q2=0.792,  F=5.74,  S=0.209.

The observed and predicted antibacterial activities of synthesized 1,3,4-oxadiazole derivatives against* Staphylococcus epidermidis* are summarized in Table 7 and plot of observed and predicted antibacterial activities is given in Figure 5.

Plot of predicted pMIC_50_ values against observed pMIC_50_ for QSAR model of all bacterial strains is given in Figure 5.

From the above mentioned QSAR models the following observations can be drawn.

- The biological activity has shown dependence on LogP, SAS, MR, ovality, MSA, and MW.
- The aromatic substituents like methoxy benzene** (6a–h)**, N-phenyl anthranilic acid** (8a–h)**, and* o-*benzoyl benzoic acid** (13a–h)** in addition to 1,3,4-oxadiazole moiety which resulted in increased lipophilicity leads to better biological activity.
- Also the presence of electron withdrawing group at para and ortho positions in the compounds resulted in enhanced biological activities.
- The results suggested that the antimicrobial activity was highly dependent on LogP, SAS, MR, ovality, MSA, and MW.
- The derived models could be used in designing of more potent inhibitors against microbial infections.
- Six parameter correlation equations for antimicrobial activity, having good values of correlation coefficient (*r* ^2^) and minimum standard error of estimate (*S*), developed against* E. coli*,* P. aeruginosa*,* S. aureus*, and* S. epidermidis*, could be used for the prediction of biological activities of unknown and unavailable compounds of this class.

### 5.4. Molecular Docking Studies

**Figure 6:**
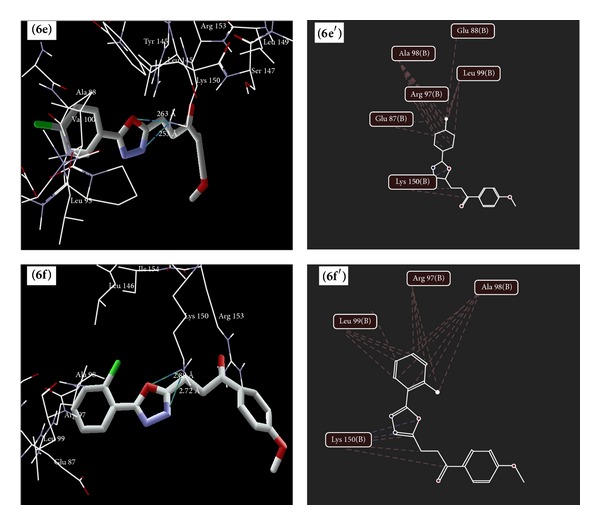
Binding modes of** 6e** and** 6f** (**6e**,** 6f** as docking view and** 6** **e**′,** 6** **f**′ as interaction view) with peptide deformylase, where blue/green lines and red lines represent hydrogen bonding and favourable steric interactions, respectively.

**Figure 7:**
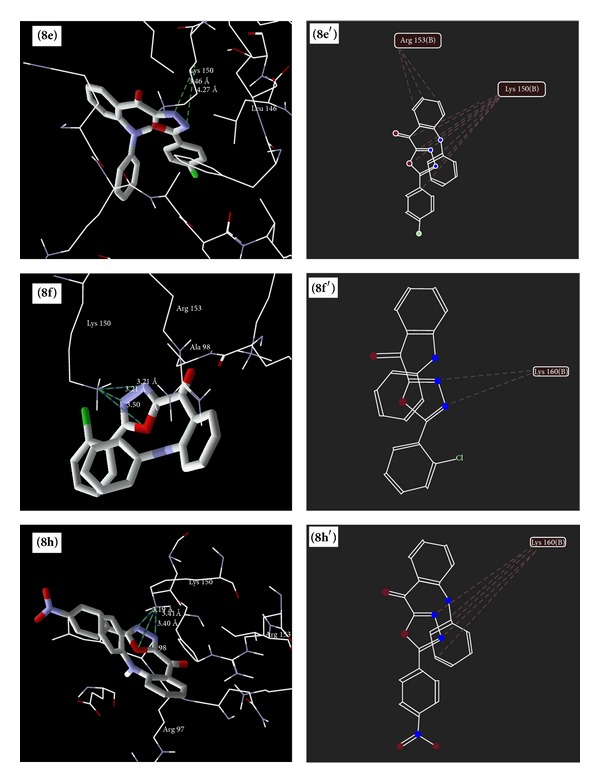
Binding modes of** 8e**,** 8f** and** 8h** (**8e**,** 8f** and** 8h** as docking views;** 8** **e**′,** 8** **f**′, and** 8** **h**′ as interaction views) with peptide deformylase, where blue/green lines and red lines represent hydrogen bonding and favourable steric interactions, respectively.

**Figure 8:**
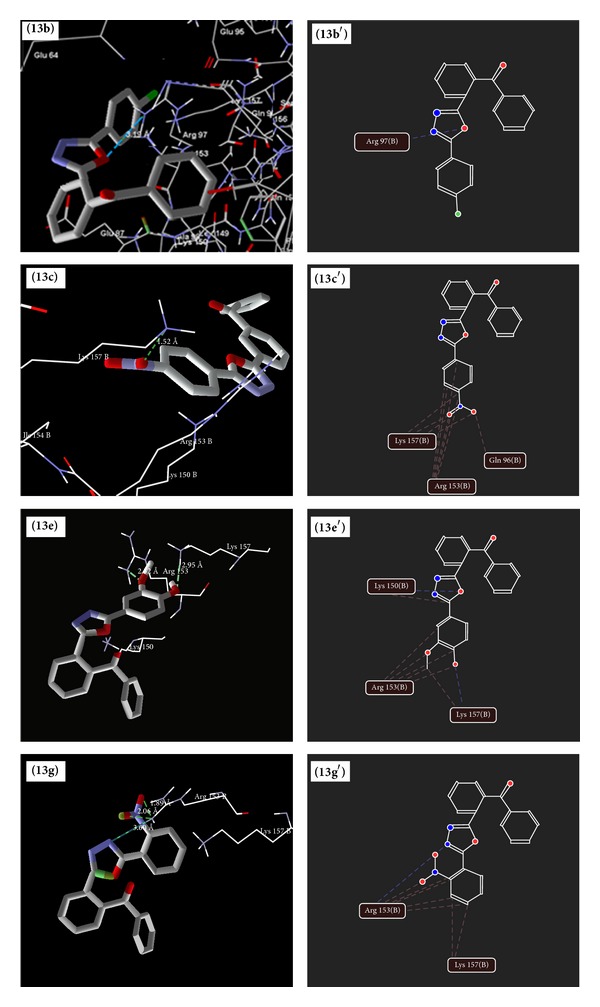
Binding modes of** 13b**,** 13c**,** 13e** and** 13g** (**13b**,** 13c**,** 13e** and** 13g** as docking views;** 13** **b**′,** 13** **c**′,** 13** **e**′ and** 13** **g**′ as interaction views) with peptide deformylase, where blue/green lines and red lines represent hydrogen bonding and favourable steric interactions, respectively.

**Figure 9:**
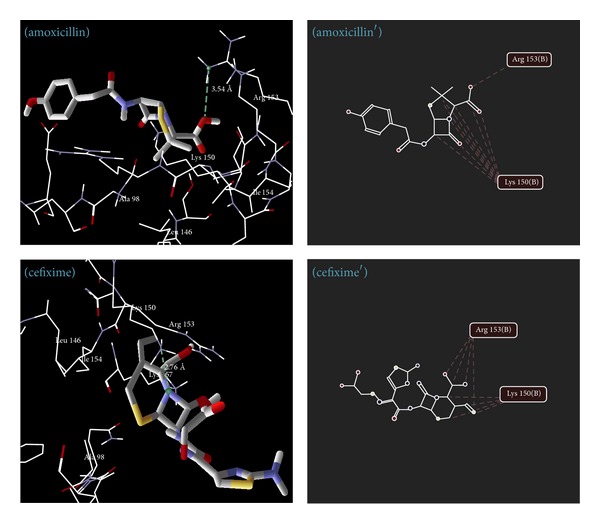
Binding modes of amoxicillin and cefixime (amoxicillin and cefixime as docking views; amoxicillin' and cefixime' as interaction views) with peptide deformylase, where blue/green lines and red lines represent hydrogen bonding and favourable steric interactions, respectively.

The results of docking study of synthesized 1,3,4-oxadiazole derivatives (Table 7) (Figures 6, 7, 8, and 9) with peptide deformylase depicted the hydrogen bonding interactions with Lys 150 (**6e**,** 6f**,** 8e**,** 8f**,** 8h**,** 13c,** and** 13e**), Lys 157 (**13e**), Arg 97 (**13b** and** 13c**), and Arg 153 (**13e** and** 13g**). The amino acids Lys 150 and Arg 153 were involved in interaction with the standard drugs (cefixime and amoxicillin) as well as the synthesized 1,3,4-oxadiazole derivatives. The docking score of derivatives** 6f**,** 8e**,** 8f**,** 8h,** and** 13e** was found to be higher than amoxicillin and** 13e** was found to have the highest dock scoring derivative than that of amoxicillin and cefixime.

In synthesized 1,3,4-oxadiazole derivatives replacement of free carboxylic group by oxadiazole nucleus enhanced the receptor interaction by formation of numerous hydrogen bonds and favourable steric interactions with peptide deformylase. These results could be used for the development of novel, potent, and effective antimicrobial agents.

The carbonyl, nitro, and 1,3,4-oxadiazole functionalities (acting as acceptor), whereas –NH– and hydroxyl group (acting as donor) in the synthesized derivatives have played very imperative position in ligand-receptor interaction for the creation of numerous hydrogen bonds. Insertion of electron withdrawing 1,3,4-oxadiazole offers an upgraded*π*-electron delocalization across the donor-acceptor links and affords significant electron transportation. Results also revealed that the hydrogen bond distance is important in docking studies. The distance more than 3.2 Å indicates feeble hydrogen bonding between ligand and receptor, 2.6 Å–3.2 Å represents virtuous hydrogen bonding, and less than 2.5 Å indicates robust bonding. Almost all the active derivatives showed good hydrogen bonding with enzymes.

## 6. Conclusion

Among all the synthesized derivatives** 6e**,** 6f**,** 13b**,** 86**,** 8f**,** 8h 13c,** and** 13e** were observed as the best antibacterial agents against all the selected microbial strains. While studying MIC against bacterial strains, compound** 13b** with* p*-chloro and** 13e** with* m*-methoxy and* p*-hydroxyl substitutions were found to be the most active among all the derivatives. In developed QSAR models, six physicochemical parameters (LogP, SAS, MR, ovality, MSA, and MW) were instituted to be important for the antibacterial activity. All the developed models have good coefficient of correlation (0.796–0.885), coefficient of determination (0.616–0.815), and cross validated *R* ^2^ (0.792–0.888) with good Fischer statistics (3.94–12.02). Among all the synthesized compounds** 13e** was found to be most potent peptide deformylase inhibitor with the highest dock score (−153.44) than that of amoxicillin and cefixime.

## Figures and Tables

**Table 1:** Substituted groups (R) of different synthesized compounds.

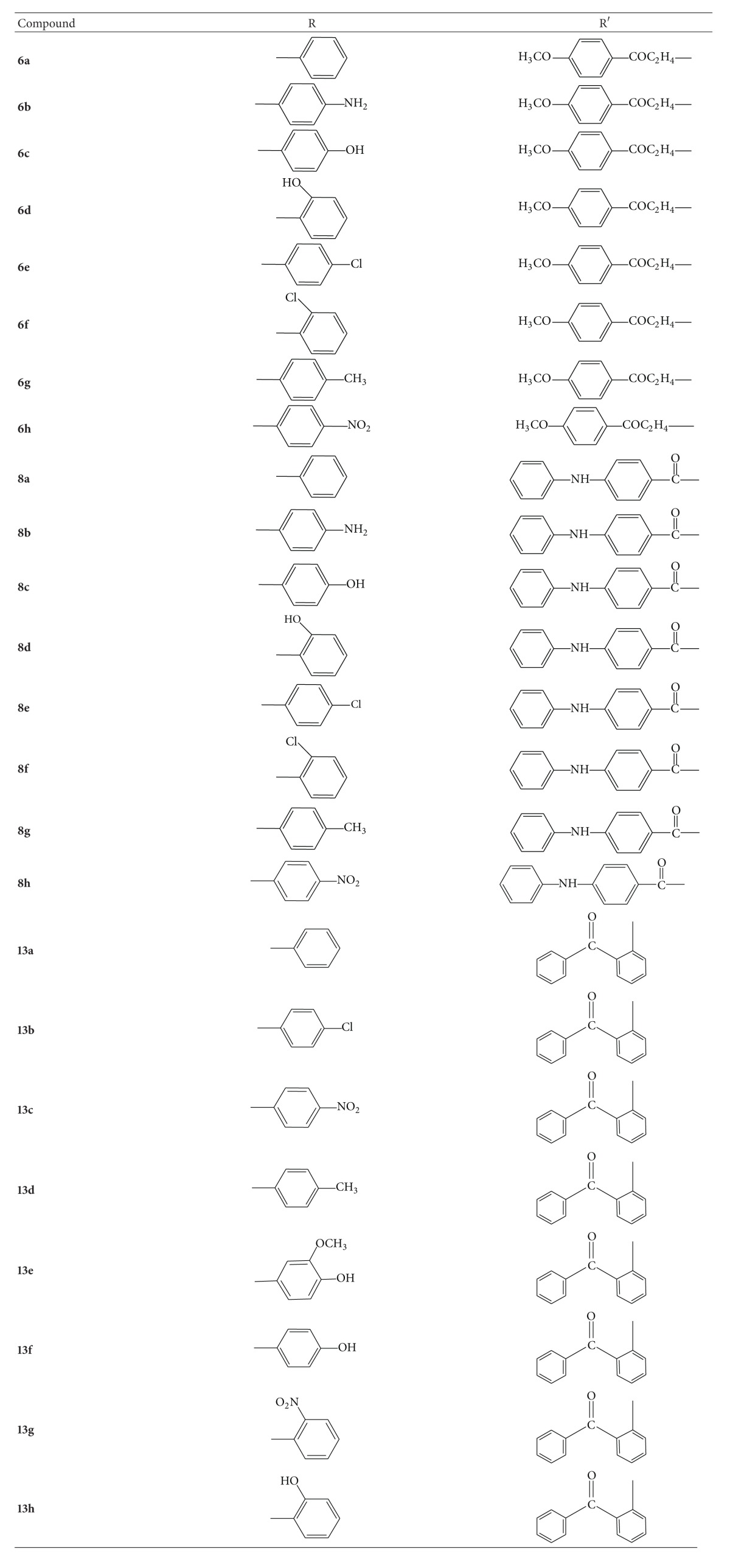

**Table 2:** Physical properties and UV-Visible analysis of synthesized 1,3,4-oxadiazole derivatives.

Compound	Molecular formula	Molecular weight	Solubility	*λ*_max⁡_	*R*_*f*_ value
**6a**	C_18_H_16_N_2_O_3_	308.33	DMSO, MeOH, CHCl_3_	263	0.54
**6b**	C_18_H_17_N_3_O_3_	323.35	DMSO, MeOH, CHCl_3_	278	0.42
**6c**	C_18_H_16_N_2_O_3_	324.33	DMSO, MeOH, CHCl_3_	265	0.47
**6d**	C_18_H_16_N_2_O_3_	324.33	DMSO, MeOH, CHCl_3_	250	0.68
**6e**	C_18_H_15_ClN_2_O_3_	342.78	DMSO, MeOH, CHCl_3_	266	0.40
**6f**	C_18_H_15_ClN_2_O_3_	342.78	DMSO, MeOH, CHCl_3_	265	0.43
**6g**	C_19_H_18_N_2_O_3_	322.36	DMSO, MeOH, CHCl_3_	254	0.41
**6h**	C_18_H_15_N_3_O_5_	353.33	DMSO, MeOH, CHCl_3_	245	0.35
**13a**	C_21_H_14_N_2_O_2_	326.35	DMSO, MeOH, CHCl_3_	281	0.51
**13b**	C_21_H_13_ClN_2_O_2_	360.79	DMSO, MeOH, CHCl_3_	281	0.58
**13c**	C_21_H_13_N_3_O_4_	371.35	DMSO, MeOH, CHCl_3_	283	0.49
**13d**	C_22_H_16_N_2_O_2_	340.37	DMSO, MeOH, CHCl_3_	281	0.55
**13e**	C_22_H_16_N_2_O_4_	372.37	DMSO, MeOH, CHCl_3_	281	0.61
**13f**	C_21_H_14_N_2_O_3_	342.35	DMSO, MeOH, CHCl_3_	285	0.60
**13g**	C_21_H_13_N_3_O_4_	371.35	DMSO, MeOH, CHCl_3_	285	0.58
**13h**	C_21_H_14_N_2_O_3_	342.35	DMSO, MeOH, CHCl_3_	283	0.67

Mobile phase for TLC: petroleum ether: ethyl acetate : MeOH (6 : 3 : 1).

**Table 3:** Zone of inhibition of synthesized 1,3,4-oxadiazole derivatives against selected microbial strains.

Compound	Concentration (*µ*g/mL)	Zone of inhibition (mm)			
		*E. coli *	*P. aeruginosa *	*S. aureus *	*S. epidermidis *
**6a**	100	16.00 ± 0.20	—	10.81 ± 0.50	12.17 ± 0.16
	200	21.80 ± 0.15	—	11.80 ± 0.40	14.98 ± 0.22
	400	21.93 ± 0.20	—	11.00 ± 0.08	15.98 ± 0.23

**6b**	100	15.90 ± 0.90	—	15.03 ± 0.10	14.01 ± 0.34
	200	18.99 ± 0.10	12.02 ± 0.18	16.01 ± 0.21	13.17 ± 0.17
	400	21.96 ± 0.17	12.12 ± 0.14	16.06 ± 0.21	18.00 ± 0.31

**6c**	100	13.11 ± 0.20	—	14.11 ± 0.11	—
	200	14.01 ± 0.02	—	17.03 ± 0.25	11.01 ± 0.13
	400	15.97 ± 0.27	—	17.06 ± 0.18	12.15 ± 0.15

**6d**	100	14.03 ± 0.19	12.03 ± 0.23	15.11 ± 0.12	—
	200	15.00 ± 0.11	14.11 ± 0.11	16.17 ± 0.17	14.07 ± 0.24
	400	16.03 ± 0.18	14.06 ± 0.25	16.71 ± 0.10	15.10 ± 0.11

**6e**	100	20.02 ± 0.09	14.12 ± 0.12	15.00 ± 0.10	16.98 ± 0.23
	200	27.04 ± 0.27	20.04 ± 0.12	23.47 ± 0.45	25.18 ± 0.23
	400	27.82 ± 0.16	20.63 ± 0.16	24.60 ± 0.11	25.86 ± 0.17

**6f**	100	15.93 ± 0.40	14.13 ± 0.11	12.05 ± 0.23	20.13 ± 0.11
	200	24.85 ± 0.14	19.91 ± 0.13	15.90 ± 0.18	24.17 ± 0.20
	400	25.94 ± 0.15	20.00 ± 0.35	16.06 ± 0.25	24.88 ± 0.13

**6g**	100	16.11 ± 0.11	—	14.12 ± 0.13	12.20 ± 0.20
	200	20.01 ± 0.19	—	18.16 ± 0.50	15.10 ± 0.13
	400	20.09 ± 0.18	—	20.21 ± 0.25	16.00 ± 0.33

**6h**	100	15.93 ± 0.06	—	15.91 ± .090	14.06 ± 0.29
	200	20.18 ± 0.50	12.05 ± 0.26	19.12 ± 0.11	13.09 ± 0.13
	400	20.80 ± 0.12	12.45 ± 0.18	22.01 ± 0.10	17.90 ± 0.24

**13a**	50	20.97 ± 0.17	18.40 ± 0.16	15.99 ± 0.14	12.91 ± 0.06
	100	21.06 ± 0.24	19.16 ± 0.50	19.96 ± 0.22	18.16 ± 0.50
	200	21.95 ± 0.24	22.20 ± 0.19	22.18 ± 0.22	19.80 ± 0.19

**13b**	50	19.89 ± 0.11	13.10 ± 0.15	22.07 ± 0.21	16.05 ± 0.31
	100	27.85 ± 0.16	17.99 ± 0.01	25.04 ± 0.27	25.03 ± 0.26
	200	28.95 ± 0.18	18.11 ± 0.11	26.16 ± 0.18	26.90 ± 0.14

**13c**	50	22.10 ± 0.09	15.91 ± 0.50	24.04 ± 0.07	20.10 ± 0.11
	100	27.05 ± 0.12	20.89 ± 0.11	26.95 ± 0.23	27.00 ± 0.17
	200	28.15 ± 0.18	22.13 ± 0.15	27.08 ± 0.24	28.00 ± 0.08

**13d**	50	14.06 ± 0.11	20.02 ± 0.18	16.01 ± 0.21	20.00 ± 0.07
	100	18.11 ± 0.10	22.80 ± 0.19	19.07 ± 0.06	26.13 ± 0.11
	200	18.09 ± 0.12	22.84 ± 0.15	19.86 ± 0.50	28.03 ± 0.14

**13e**	50	24.02 ± 0.12	16.00 ± 0.02	24.01 ± 0.28	24.00 ± 0.20
	100	27.15 ± 0.18	21.00 ± 0.18	27.05 ± 0.33	26.00 ± 0.17
	200	28.01 ± 0.18	22.17 ± 0.20	28.34 ± 0.24	28.08 ± 0.07

**13f**	50	18.13 ± 0.33	16.11 ± 0.11	14.84 ± 0.19	11.81 ± 0.15
	100	20.10 ± 0.10	21.97 ± 0.18	14.84 ± 0.19	18.03 ± 0.12
	200	20.13 ± 0.15	21.90 ± 0.20	15.98 ± 0.27	18.16 ± 0.19

**13g**	50	14.12 ± 0.13	11.91 ± 0.90	16.05 ± 0.26	16.00 ± 0.22
	100	24.02 ± 0.14	15.21 ± 0.25	19.90 ± 0.06	26.14 ± 0.15
	200	25.86 ± 0.15	15.95 ± 0.06	19.98 ± 0.13	27.91 ± 0.08

**13h**	50	20.03 ± 0.15	11.10 ± 0.09	14.01 ± 0.32	19.80 ± 0.19
	100	21.14 ± 0.15	12.90 ± 0.15	16.34 ± 0.24	22.03 ± 0.34
	200	21.05 ± 0.24	13.00 ± 0.17	16.98 ± 0.17	23.09 ± 0.09

Amoxicillin	200	22.00 ± 0.67	—	10.98 ± 0.10	24.09 ± 0.10
	400	26.20 ± 0.44	12.11 ± 0.11	14.19 ± 0.18	27.99 ± 0.10
	800	30.00 ± 0.10	13.99 ± 0.23	18.12 ± 0.12	32.06 ± 0.22

Cefixime	200	16.00 ± 0.28	—	—	14.88 ± 0.12
	400	18.15 ± 0.17	—	—	15.99 ± 0.30
	800	22.18 ± 0.22	12.00 ± 0.38	12.03 ± 0.19	20.03 ± 0.15

Values are expressed as mean ± standard deviation of the three replicates.

Diameter of the well is not included in zone of inhibition.

**Table 4:** Minimum inhibitory concentrations (MIC) of synthesized 1,3,4-oxadiazole derivatives.

Compound	MIC (*µ*g/mL)					
	*E. coli *	*P. aeruginosa *	*S. aureus *	*S. epidermidis *	*C. albicans *	*A. niger *
**6a**	50	100	50	200	50	100
**6b**	50	200	50	100	25	200
**6c**	25	200	100	100	200	50
**6d**	200	200	100	200	200	100
**6e**	25	100	25	100	200	50
**6f**	12.5	100	100	12.5	12.5	25
**6g**	50	100	100	50	25	25
**6h**	25	100	50	25	50	200
**13a**	25	200	25	50	50	50
**13b**	12.5	12.5	100	12.5	12.5	10
**13c**	25	25	100	25	25	10
**13d**	25	25	50	50	50	50
**13e**	12.5	25	25	25	100	10
**13f**	50	50	100	25	25	25
**13g**	50	25	50	50	50	100
**13h**	50	100	200	25	100	200
Control	—	—	—	—	—	—
Amoxicillin	12.5	200	100	12.5	—	—
Cefixime	50	400	400	50	—	—
Fluconazole	—	—	—	—	12.5	400

**Table 5:** Observed and predicted antibacterial activity of synthesized 1,3,4-oxadiazole derivatives against *Escherichia coli* and *Pseudomonas aeruginosa*.

Compounds	Observed activity	Calculated activity	Residuals	Observed activity	Calculated activity	Residuals
**6a**	1.39794	1.42696	−0.02902	1.39794	1.380355	0.017585
**6b**	1.39794	1.49081	−0.09287	—	—	—
**6c**		—	—	1.69897	1.693166	0.005804
**6d**	—	—	—	1.69897	1.749717	−0.05075
**6e**	1.09691	1.17324	−0.07633	—	—	—
**6f**	—	—	—	1.69897	1.739458	−0.04049
**6g**	1.39794	1.28492	0.113024	1.69897	1.565732	0.133238
**8a**	1.09691	1.10285	−0.00594	—	—	—
**8b**	1.09691	1.22329	−0.12638	—	—	—
**8c**	1.09691	1.01685	0.080062	—	—	—
**8d**	2	1.95106	0.048936	2	2.143298	−0.1433
**8e**	—	—	—	—	—	—
**8f**	1.69897	1.66784	0.031132	—	—	—
**8g**	1.09691	1.0879	0.009007	1.39794	1.417768	−0.01983
**13a**	1.09691	1.2223	−0.12539	1.09691	1.241458	−0.14455
**13b**	0.79588	0.9957	−0.19982	1.69897	1.59415	0.10482
**13d**	1.09691	1.13534	−0.03843	1.39794	1.422187	−0.02425
**13e**	0.79588	0.80956	−0.01368	—	—	—
**13f**	1.39794	1.03279	0.365149	1.69897	1.642724	0.056246
**13h**	1.09691	1.11426	−0.01735	2	1.769982	0.230018

(—) Compounds are not included in QSAR model development.

**Table 6:** Observed and predicted antibacterial activities of 1,3,4-oxadiazole derivatives against *Staphylococcus aureus* and *Staphylococcus epidermidis*.

Compounds	*Staphylococcus aureus *			*Staphylococcus epidermidis *		
	Observed activity	Calculated activity	Residuals	Observed activity	Calculated activity	Residuals
**6a**	1.69897	1.747218	−0.04825	2	2.018882	−0.018880
**6b**	2	2.061071	−0.06107	1.69897	1.726729	−0.027760
**6c**	2	1.875044	0.124956	1.69897	1.573922	0.125048
**6d**	2	1.834783	0.165217	—	—	—
**6e**	1.69897	2.002453	−0.30348	1.69897	1.740717	−0.041750
**6f**	1.69897	1.827713	−0.12874	—	—	—
**6g**	1.69897	1.462069	0.236901	—	—	—
**8a**	—	—	—	—	—	—
**8b**	1.09691	1.17198	−0.07507	1.69897	1.591267	0.107703
**8c**	1.09691	1.151688	−0.05478	1.69897	1.732577	−0.033610
**8d**	2	1.808758	0.191242	1.39794	1.343779	0.054161
**8e**	2	1.885819	0.114181	1.09691	0.887520	0.209390
**8f**	1.69897	1.552991	0.145979	1.39794	1.353518	0.044422
**8g**	1.09691	1.268834	−0.17192	1.39794	1.327929	0.070011
**13a**	—	—	—	1.39794	1.308439	0.089501
**13b**	—	—	—	0.79588	1.030457	−0.23458
**13d**	1.09691	1.056386	0.040524	1.39794	1.425514	−0.02757
**13e**	—	—	—	1.09691	1.227397	−0.13049
**13f**	1.39794	1.647697	−0.24976	1.09691	1.118010	−0.02110
**13h**	1.69897	1.611633	0.087337	1.09691	1.165225	−0.06832

(—) Compounds are not included in QSAR model development.

**Table 7:** Ligand-receptor interaction of synthesized 1,3,4-oxadiazole derivatives with peptide deformylase.

Compound	Docking score (binding energy)	Distance (Å)	Amino acid	Group involved in interaction with receptor
**6e**	−77.213	2.63	Lys 150	Oxygen of oxadiazole ring
		2.53	Lys 150	Nitrogen of oxadiazole ring
**6f**	−135.787	2.72	Lys 150	Nitrogen of oxadiazole ring
		2.88	Lys 150	Oxygen of oxadiazole ring
**8e**	−146.825	3.46	Lys 150	Nitrogen of oxadiazole ring
		4.27	Lys 150	Nitrogen of oxadiazole ring
**8f**	−138.439	3.50	Lys 150	Oxygen of oxadiazole ring
		3.21	Lys 150	Nitrogen of oxadiazole ring
		3.21	Lys 150	Nitrogen of oxadiazole ring
**8h**	−151.632	3.40	Lys 150	Oxygen of oxadiazole ring
		3.41	Lys 150	Nitrogen of oxadiazole ring
		3.19	Lys 150	Nitrogen of oxadiazole ring
**13b**	−88.30	3.19	Arg 97	Oxygen of oxadiazole ring
**13c**	−96.604	3.31	Lys 150	Nitrogen of oxadiazole ring
		3.49	Lys 150	Oxygen of oxadiazole ring
		2.69	Arg 97	Nitrogen of nitro group
**13e**	−153.44	2.95	Lys 157	Oxygen of hydroxyl group
		2.02	Arg 153	Oxygen of methoxy group
		3.26	Lys 150	Oxygen of oxadiazole ring
**13g**	−81.702	2.06	Arg 153	Oxygen of nitro group
		3.05	Arg 153	Oxygen of oxadiazole ring
Amoxicillin	−128.027	3.54	Arg 153	Oxygen of –COOH group
Cefixime	−153.288	2.76	Lys 150	Nitrogen of *β*-lactam
